# Macrophages exposed to HIV viral protein disrupt lung epithelial cell integrity and mitochondrial bioenergetics via exosomal microRNA shuttling

**DOI:** 10.1038/s41419-019-1803-y

**Published:** 2019-08-02

**Authors:** Zhihong Yuan, Jessica R. Petree, F. Eun-Hyung Lee, Xian Fan, Khalid Salaita, David M. Guidot, Ruxana T. Sadikot

**Affiliations:** 1 Department of Veterans Affairs, Atlanta VAMC, Decatur, GA 30033 USA; 2 Division of Pulmonary, Allergy, Critical Care and Sleep Medicine, Emory University School of Medicine, Atlanta, GA 30322 USA; 3 Wallace H. Coulter Department of Biomedical Engineering, Georgia Institute of Technology and Emory University, Atlanta, GA 30322 USA

**Keywords:** Monocytes and macrophages, Monocytes and macrophages, HIV infections, HIV infections

## Abstract

Antiretroviral therapy extends survival but does not eliminate HIV from its cellular reservoirs. Between immune and stromal cells in the tissue microenvironment, a dynamic intercellular communication might influence host viral immune responses via intercellular transfer of extracellular vehicles (EVs) (microvesicles, exosome, or apoptotic bodies). It is increasingly recognized that HIV-infected macrophage-secreted nucleotide-rich exosomes might play a critical role in mediating communication between macrophages and other structural cells; however, molecular mechanisms underlying cell–cell crosstalk remain unknown. Here we show that HIV-1-infected macrophages and HIV-1 proteins Tat or gp120-treated macrophages express high levels of microRNAs, including miR-23a and miR-27a. Identical miRNAs expression patterns were detected in macrophage-secreted exosomes isolated from bronchoalveolar lavage fluid of HIV transgenic rats. Tat-treated macrophage-derived exosomal miR-23a attenuated posttranscriptional modulation of key tight junction protein zonula occludens (ZO-1) 3′-UTR in epithelial cells. In parallel, exosomal miR-27a released from Tat-treated macrophages altered the mitochondrial bioenergetics of recipient lung epithelial cells by targeting peroxisome proliferator-activated receptor gamma (PPARγ), while simultaneously stimulating glycolysis. Together, exosomal miRNAs shuttle from macrophages to epithelial cells and thereby explain in part HIV-mediated lung epithelial barrier dysfunction. These studies suggest that targeting miRNAs may be of therapeutic value to enhance lung health in HIV.

## Introduction

The human immunodeficiency virus (HIV) pandemic continues to be a major global challenge, despite the widespread availability of antiretroviral drugs. Although the pandemic appears to be stabilizing, there are ~34 million people living with HIV worldwide. The development of combined antiretroviral therapy (ART) has been effective in extending the lives of people infected with HIV and curbing the spread of disease. Even when adherent to ART, individuals with HIV are at greater risk for a variety of lung infections and other lung diseases^1,2^. The lung epithelium constitutes the first line of host defense against invading pathogens^3^. HIV can infect macrophages and thereby impair key defense mechanisms such as phagocytosis and bacterial killing^4–6^. Exosomes released by HIV-infected macrophages might influence the lung tissue microenvironment via transcellular delivery to other structural cells, such as lung epithelial cells and endothelial cells. Lung epithelial cells can take up extracellular vesicles released by macrophages with consequent dysregulated inflammatory signaling and immune function^7,8^. However, if this exchange of exosomes between immune and structural cells occurs in HIV infection and influences cell functions, mechanisms have not been investigated.

Exosomes are 20–200 nm diameter extracellular vesicles secreted from late endosomes from multiple types of cells, including immune and stromal cells, such as endothelial cells, epithelial cells, and tumor cells^9^. Exosomes are important mediators of intercellular communication in both physiological and pathophysiological states^10–12^. Exosomes can carry various proteins, lipids, mRNA, metabolic enzymes, and microRNAs (miRNAs), which can regulate cellular processes through transcellular delivery of molecular content^13–15^. MiRNAs are 18–22-nucleotide noncoding RNA molecules that regulate gene expression posttranscriptionally through base pairing with mRNAs, resulting in either translational repression or mRNA degradation, and thus regulate the expression of multiple genes at a posttranscriptional level^16,17^.

Exosomal miRNAs have shown to be modulators in variety of diseases, including cancer, infections, obesity, diabetes, and cardiovascular disease^18–24^. Via facilitating cell-to-cell communication, exosomes can bind to recipient cells and deliver packaged contents, including mRNA, miRNAs, and proteins capable of influencing recipient cells^25,26^. Emerging data suggest that exosomes may be involved in HIV-1 pathogenesis^27–30^. In the alveolar space, cell–cell interactions between alveolar macrophages and epithelial cells can influence host defense and innate immunity^11^. However, the molecular mechanisms underlying the effects of HIV-infected macrophage-derived exosomal miRNAs on other host cells remain to be defined.

In this study we show that miR-23a ~ 27a~ 24 cluster is significantly upregulated and secreted by macrophages exposed to HIV viral proteins and modulates epithelial barrier integrity through dysregulation of tight junction protein expression and perturb mitochondrial function in lung epithelial cells. These studies provide novel evidences that macrophage-derived exosomal miRNAs can facilitate cell–cell communications in the alveolar microenvironment, which in turn impair lung health in these vulnerable individuals living with chronic HIV infection.

## Results

### HIV-related viral proteins induce expression of select miRNAs in macrophages

**Fig. 1:**
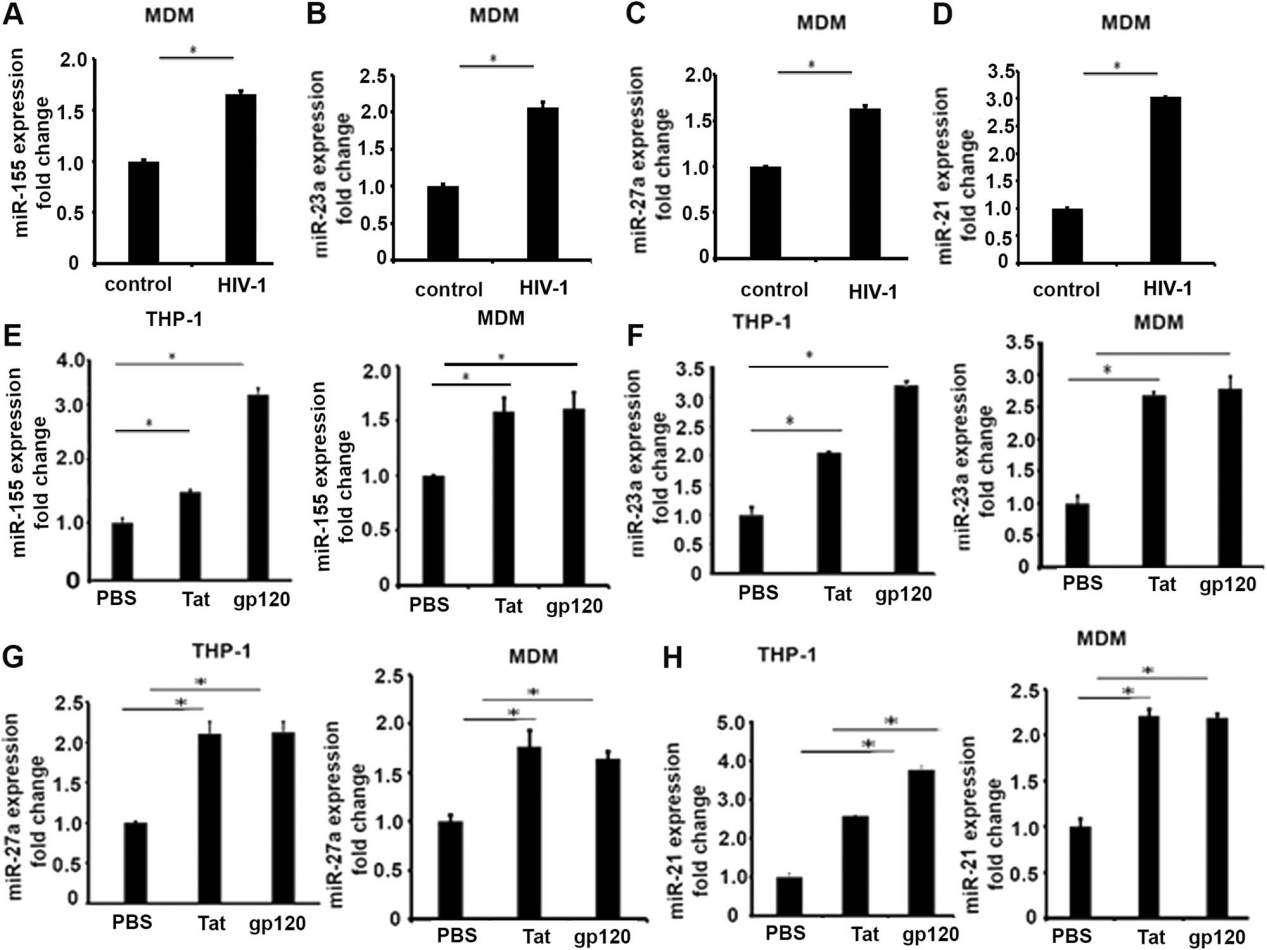
Expression of select miRNAs were induced in HIV-1-infected human monocyte-derived macrophages (MDMs) and recombinant HIV viral protein-treated MDMs. Human MDMs were infected with HIV-1 for 8 days. MiRNAs were extracted and analyzed via qRT-PCR and normalized to U6 gene. **a** miR-155; **b** miR-23a; **c** miR-27a; **d** miR-21. Student’s test, *p < 0.05. THP-1-derived macrophages and human MDMs were treated with recombinant HIV viral protein Tat (100 ng/ml) and gp120 (100 ng/ml), respectively. Twenty-four hours later, expression levels of miRNAs were analyzed by qRT-PCR and normalized to U6 gene. **e** miR-155; **f** miR-23a; **g** miR-27a; **h** miR-21. Student’s test, *p < 0.05

Recent studies have shown that miRNA expression in HIV-infected immune cells affect viral infection and latency^31^. In our previous studies, microarray profile showed expression of a couple of miRNAs is associated with inflammatory responses, including miR-155, miR-21, and members of miR-23a~ 27a~ 24 cluster, such as miR-23a and miR-27a^32^. We first determined whether HIV-1-infected human macrophages express the key miRNAs modulating inflammatory responses. Human monocyte-derived macrophages (MDMs) were infected with HIV-1 as described by us previously^33^. We found that expression of miR-155, miR-23a, miR-27a, and miR-21 was significantly increased by HIV-1 infection, compared with uninfected cells, as shown in Fig. 1a–d.

Then we determined whether HIV viral proteins gp120 and Tat alter expression of selective miRNAs. THP-1-derived macrophages and human MDMs with Tat and gp120 (100 ng/ml), separately, and detected intracellular miRNAs expression. Quantitative reverse-transcriptase PCR (qRT-PCR) results showed that expression of miR-155, miR-21, miR-23a, and miR-27a were significantly upregulated in both viral protein-treated macrophages, compared with the phosphate-buffered saline (PBS) control group (Fig. 1e–h). Together, these data suggest that HIV infection and HIV viral proteins induce expression of selective miRNA, which can contribute to inflammatory and oxidant responses, which may impact the overall immune response.

### Macrophages exposed to HIV-related proteins secrete exosomes containing miRNAs

**Fig. 2:**
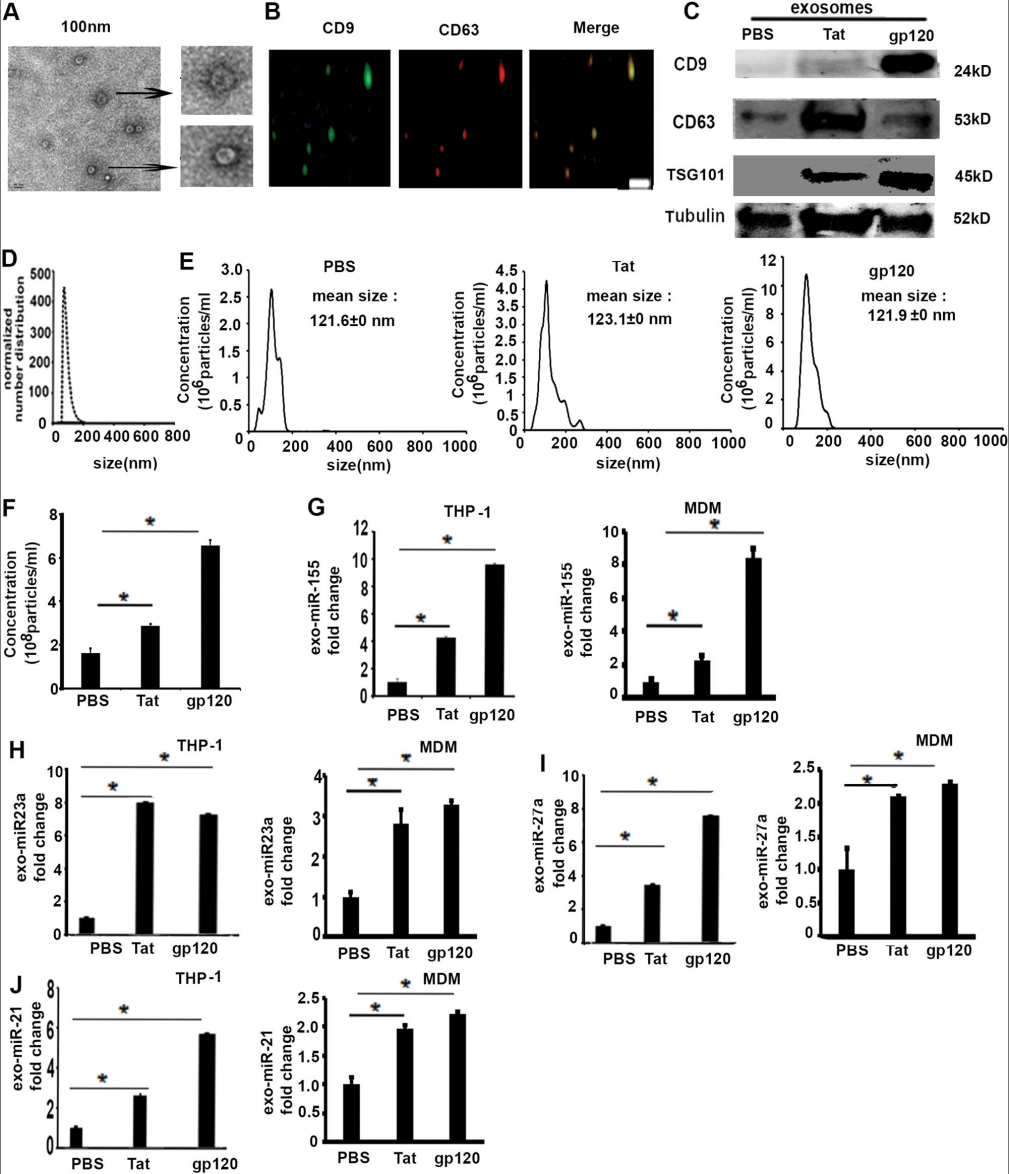
Macrophages exposed to HIV viral proteins secrete exosomes containing miRNAs. Exosomes were isolated from cell culture media of THP-1-derived macrophages and MDM. **a** Representative transmission electron microscopy (TEM) images showing exosomes, scale bar: 100 nm. **b** Representative immunofluorescence images of exosomes stained with antibodies anti-CD9 and anti-CD63. Scale bar: 10 µm. **c** Western blotting showing surface markers CD9, CD63, and TSG101 of exosomes secreted by macrophages exposed to Tat (100 ng/ml) and gp120 (100 ng/ml) for 24 h. Tubulin serves as internal control. Analysis of the size distribution and particles concentration of human macrophage-secreted exosome fractions using DLS (**d**) and Nanoparticle Tracking Analysis (NTA) (**e**). **f** Quantification of exosomes secreted by NDM exposed to Tat (100 ng/mL) or gp120 (100 ng/mL). PBS was used as control. Student’s test, *p *<* 0.05. **g**–**j** Exosomal miRNAs were isolated from exosomes secreted by human macrophages exposed to HIV viral proteins Tat or gp120, as well as PBS, then expression level of miR-155, miR-23a, miR-27a, and miR-21 were measured by qRT-PCR normalized to U6 SnRNA expression, *n* *=* 4–5, Student’s test, *p *<* 0.05

Recent studies show that exosomal miRNA expression levels are significantly increased in patients’ sera with HIV, which can modulate interaction between HIV-infected immune cells in lung microenvironment^34^. Exosomal miRNAs might play a pivotal role in intercellular communication in lung diseases. As we found increased expression of miRNAs in macrophages treated with HIV viral proteins, we hypothesized that macrophages expressing HIV-related proteins could influence neighboring cells via secretion of exosomes as vehicles for intercellular communication^35^. To validate this, first we isolated exosomes from the cultured media of THP-1-derived macrophages treated with HIV viral proteins, as described in the Materials and Methods. The representative image of exosomes was captured by transmission electron microscopy (TEM), as shown in Fig. 2a. Exosomal fractions were examined using both immunofluorescence staining and western blotting analysis for surface exosome markers^36^. Isolated exosomes from HIV-treated proteins were characterized by immunofluorescence staining with anti-CD9 and anti-CD63 antibodies (Fig. 2b). Western blotting analysis further confirmed that exosomes contained specific antigens, including CD9, CD63, and TSG101 (Fig. 2c). To determine the size distribution of exosomes released from cultured cells, a nanoparticle analyzer was used to track the released exosomes. Dynamic light scattering (DLS) analysis showed that the peak diameter of released exosomes is around 100 nm (Fig. 2d). Human macrophages were exposed to Tat or gp120, then the number of exosomes per cell section was quantified by Nanosight technology Nanoparticle Tracking Analysis (NTA) (Fig. 2e). We found that HIV protein Tat and gp120 induces an increase of exosomes numbers, compared with PBS group, as shown in Fig. 2f.

We next determined whether the exosomes released from macrophages contain the miRNAs that showed an increased expression in response to HIV viral proteins. We determined the expression within the secreted exosome expression of key miRNAs that impact inflammatory and antioxidant responses, specifically miR-155, miR-23a, miR-27a, and miR-21. As shown in Fig. 2g–j, once THP-1-derived macrophages and human MDMs were exposed to Tat or gp120, both release exosomes containing these miRNAs. These novel findings suggest that alveolar macrophages in HIV infection can secrete miRNAs in exosomes, which may alter the function of other cell types within the lung alveolar microenvironment.

### miRNA expression changes seen during exposure to HIV-related viral proteins in macrophages are reflected in lungs of HIV transgenic rats

**Fig. 3:**
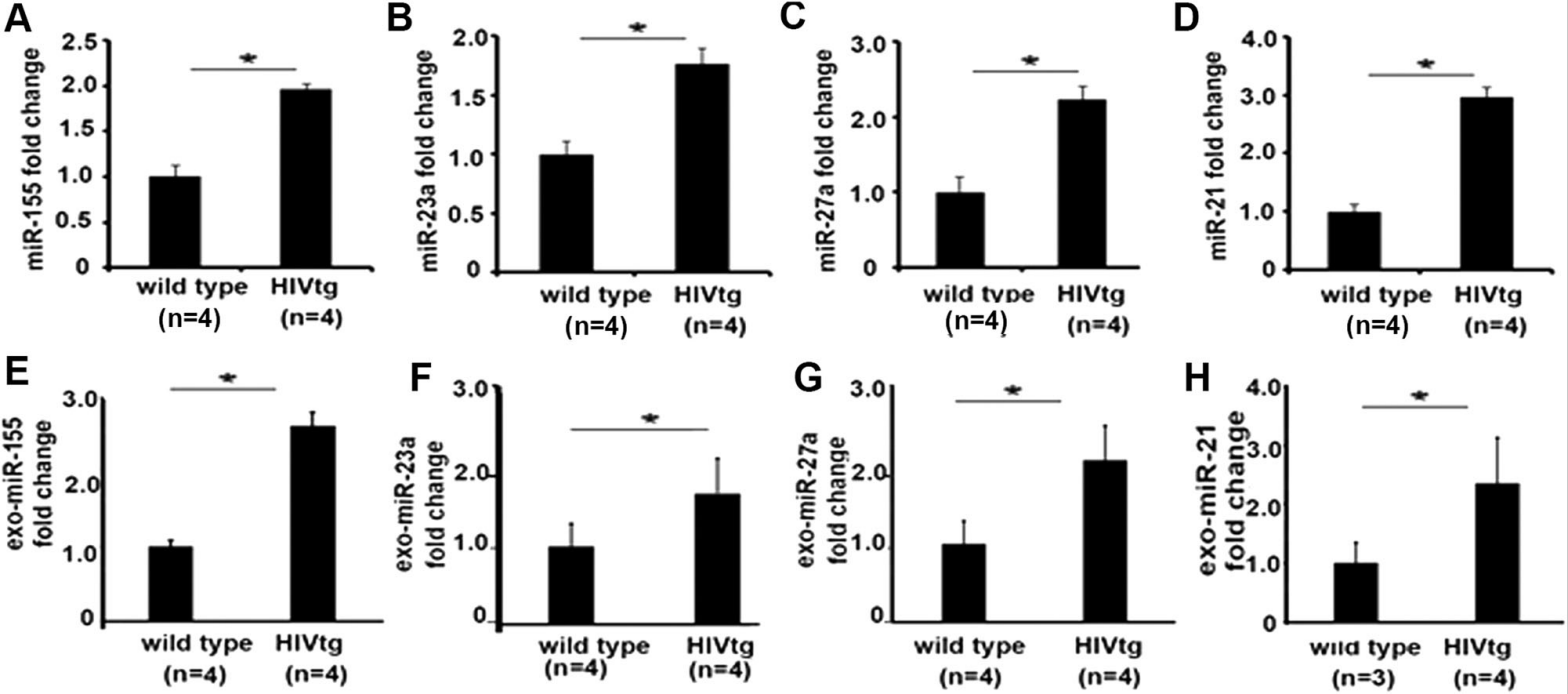
miRNA expression changes during exposure to HIV viral proteins in macrophages are reflected in lungs of HIV transgenic rats. AM were isolated from HIV transgenic and wild-type rats; expression levels of microRNAs were measured by qRT-PCR, normalized to U6. **a** miR-155; **b** miR-23a; **c** miR-27a; **d** miR-21 (*n* = 4 per group). *p *<* 0.05. Student’s test. Exosomes were isolated from BAL fluid of HIV transgenic or wild-type rats; exosomal miRNAs were isolated and detected by qRT-PCR, normalized to U6 snRNA (*n* = 4 per group). **e** miR-155; **f** miR-23a; **g** miR-27a; **h** miR-21. *p *<* 0.05. Student’s test

To determine whether an altered exosomal miRNA profile occurs in an in vivo HIV small animal model, HIV-1 transgenic rats (HIV-Tg) were used to determine exosomal miRNA expression in the lung alveolar microenvironment^37^. Alveolar macrophages and bronchoalveolar lavage (BAL) fluid were isolated from HIV transgenic and wild-type (WT) rats, respectively. Comparative quantitative miRNA fold change showed that miR-27a, miR-23a, miR-155, and miR-21 were significantly increased in alveolar macrophages of HIV transgenic rats compared with WT rats (Fig. 3a–d). In parallel, these miRNAs were also secreted in exosomes isolated from Bronchoalveolar lavage fluid (BALF) from HIV transgenic rats and were significantly increased compared with exosomes isolated from WT rats (Fig. 3e–h). Together, these data show that chronic exposure to HIV proteins induces expression of miRNAs in alveolar macrophages, which are secreted in alveolar space, and may thus impact the function of neighboring structural cells in the lung.

### Exosomal miR-23a secreted by macrophages treated with HIV viral protein impairs tight junction barrier integrity in lung epithelial cells

Next, we wanted to investigate the functional impact of the exosomal miRNAs secreted by macrophages exposed to HIV viral proteins. Previous studies have shown that expression of miR-155 and miR-21 contribute to HIV latency of macrophages and impact the inflammatory response. However, the function of miR-23a and miR-27a in HIV-infected macrophages has not been studied. Therefore, we further investigated the role of both miRNAs in HIV-infected cells.

Previously, we and others have shown that lung epithelial barrier integrity is disrupted by HIV infection^38,39^. We questioned whether the disruption of barrier function was related to secretion of miRNAs by HIV-exposed macrophages. By employing bioinformatic analysis (microRNA.org, Targetscan, and pTAR), we found that miR-23a targets tight junction protein zonula occludens (ZO-1) through binding to its 3′-untranslated region (UTR). Therefore, we investigated whether exosomal miR-23a released from macrophages exposed to HIV viral proteins contributes to barrier disruption of neighboring epithelial cells.

**Fig. 4:**
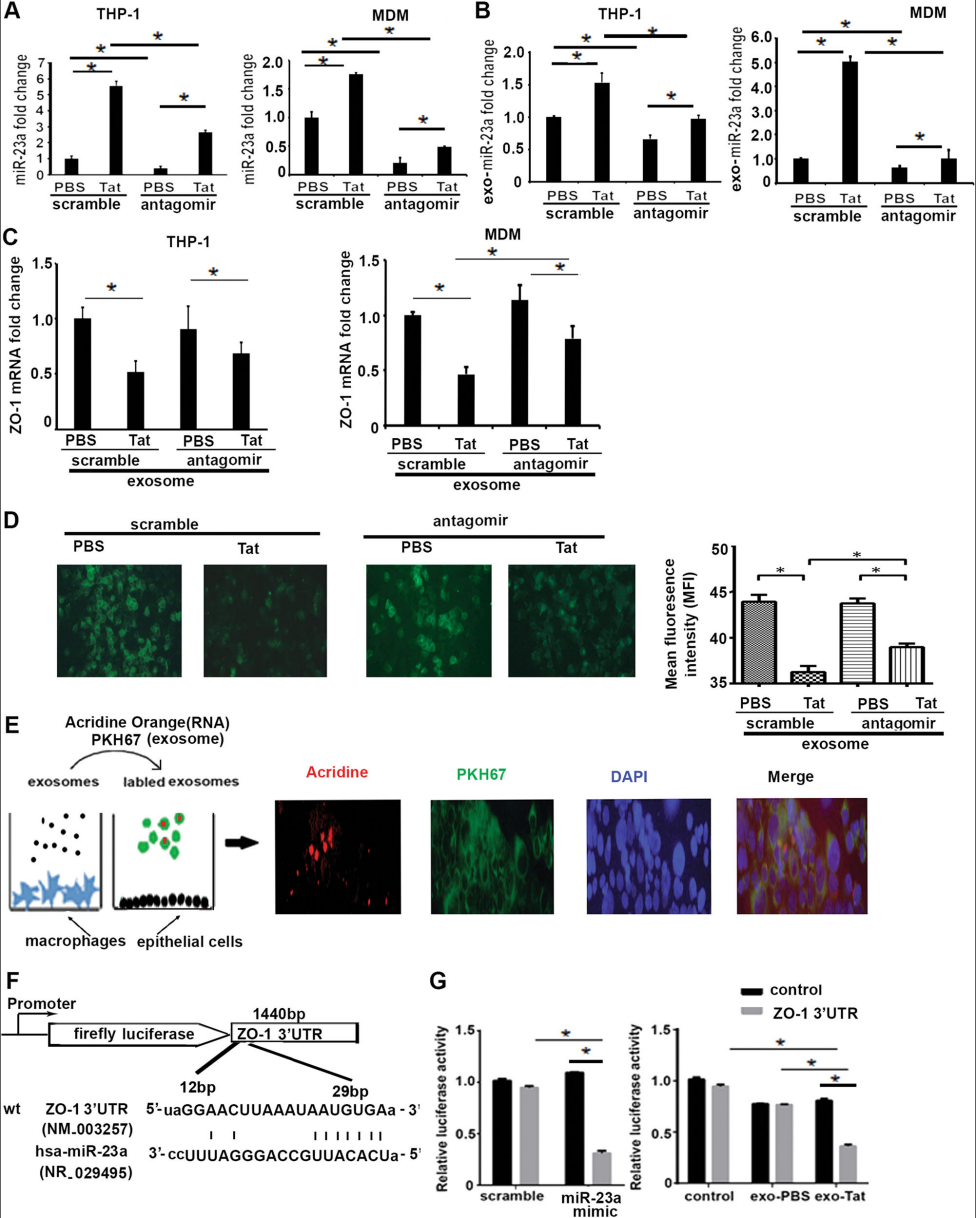
Exosomal miR-23a secreted by macrophages exposed to HIV viral protein impair expression of tight junction proteins in recipient epithelial cells. THP-1-derived macrophages and MDMs were transfected with miR-23a antagomir and Cy3-dye-labeled anti-miR-negative inhibitor control, respectively; then, they were treated with Tat (100 ng/ml) or PBS. Total miRNAs were isolated and intracellular miR-23a expression was measured with qRT-PCR. *S*tudent’s test, *p *<* 0.05 (**a**); in parallel, exosomes were isolated from cell culture supernatant, then exosomal miRNAs were isolated and miR-23a expression was analyzed by qRT-PCR. Student’s test, *p *<* 0.05 (**b**). **c** Exosomes from human macrophages were co-cultured with BEAS-2B cells, total RNA was isolated, and ZO-1 mRNA expression was measured with qRT-PCR, *p *<* 0.05. BEAS-2B cells were stained with ZO-1 antibody and representative immunofluorescence image as shown in **d**; scale bar 50 µm. Level of Fluo-488 fluorescence in BEAS-2B cells was quantified using Image J software. Mean ± SEM are provided (*n* = 3). *p *<* 0.05. Exosomes from macrophages were labeled with PKH67 and Acridine Orange, then co-cultured with BEAS-2B cells (**e**). The schematic demonstrates putative miR-23a-binding sites within the 3′-UTR of ZO-1 gene (**f**). Relative luciferase activity from pMirTarget empty vector and ZO-1 3′-UTR reporter co-transfected in BEAS-2B cells with exosomes derived from human macrophages treated with miR-23a mimic or control vector; ZO-1 luciferase activity from epithelial cells treated with exosomes derived from PBS- or Tat-treated cells (**g**). One-way ANOVA followed by the Dunn’s post hoc test was used to determine the statistical significance between multiple groups. *p *<* 0.05

We first established that antagomir inhibits miR-23a expression in macrophages and exosomes released by macrophages exposed to HIV Tat protein. Macrophages exposed to Tat showed an increased expression of cellular and exosomal miR-23a, which was significantly inhibited by miR-23a antagomir, compared with control antagomir (Fig. 4a, b). Coculture is commonly used in exploring cell–cell interaction. As shown in Supplementary Fig. S1A–C, BEAS-2B cells that co-cultured with macrophages exposed to Tat showed a decreased expression of tight junction protein ZO-1. Next, we sought to determine the role of exosomal miR-23a from macrophages on lung epithelial cells. We found that exosomes from macrophages exposed to Tat showed a significant reduction in expression of ZO-1 mRNA compared with exosomes from PBS-treated macrophages. To determine whether the reduction in the ZO-1 expression was related to miR-23a, we transfected BEAS-2B cells with antagomir of miR-23a and control antagomir. Treatment with antagomir miR-23a rescued the expression of ZO-1 in BEAS-2B cells exposed to exosomes from Tat-treated macrophages, suggesting that the reduction of ZO-1 expression was indeed related to secretion of exosomal miR-23a (Fig. 4c). Similar findings were confirmed by immunofluorescence imaging, which showed that recipient epithelial cells that were treated with exosomes from Tat-treated macrophages showed reduced expression of ZO-1, which was rescued by miR-23a antagomir (Fig. 4d). We also confirmed that exosomes released from macrophages are taken up by epithelial cells. PKH67 was used for exosomes labeling; simultaneously, Acridine orange can bind to nucleotide acids. BEAS-2B cells were co-cultured with purified PKH67 and Acridine orange-labeled exosomes derived from human macrophages; then, fluorescence microscopy was used to demonstrate that labeled exosomes harboring RNA are taken up by recipient epithelial cells (Fig. 4e).

To further confirm our findings and impact of exosomal miR-23a on epithelial tight junction protein ZO-1, we used a luciferase reporter assay. Based on computational predictions for the binding sites of miR-23a on 3′-UTR of human ZO-1 (Fig. 4f), we determined whether exosomal miR-23a affects luciferase activity of the target gene. Transfection efficacy of miRNA mimic was ~60%, as shown in Supplementary Fig. S2. Transfection of miR-23a mimic and exosomes of Tat-treated macrophages reduced luciferase activity of ZO-1 3′-UTR in lung epithelial cells, compared with exosomes from control-treated cells and mimics, respectively (Fig. 4g). Together, these findings indicate that exosomal miR-23a released from macrophages exposed to HIV viral proteins can disrupt epithelial integrity of neighboring epithelial cells by reducing the expression of tight junction proteins.

### Mitochondrial dysfunction in lung epithelial cells caused by exosomal miR-27a secreted by HIV-treated macrophages

**Fig. 5:**
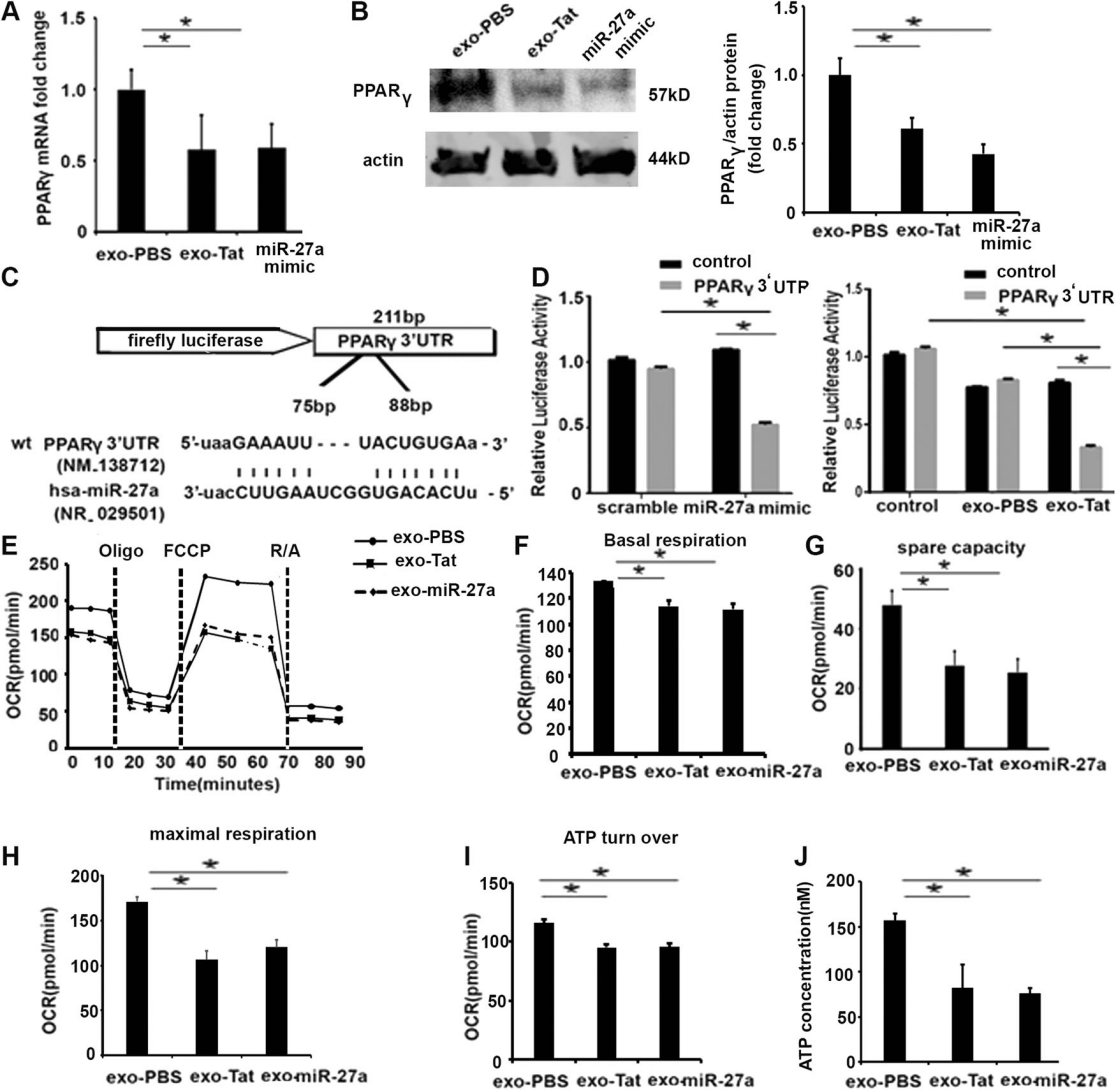
Exosomal miRNA-27a secreted by macrophages exposed to HIV protein disrupts mitochondrial bioenergetics through suppressing OCR. BEAS-2B cells were transfected with exosomes derived from macrophages treated with HIV Tat protein, control cells, and miR-27a mimics or exo-miR-23a mimics. Expression level of target genes was measured by qRT-PCR and western blotting; **a** fold changes of PPARγ mRNA; **b** expression of PPARγ protein; **c** schematic map showing putative miR-27a-binding site on the 3′-UTR of PPARγ gene; **d** relative luciferase activity from pMirTarget empty vector and PPARγ 3′-UTR reporter transfected in BEAS-2B epithelial cells treated with exosomes derived from human macrophages treated with scramble of miR-27a mimic. *p *<* 0.05. PPARγ 3′-UTR reporter transfected in BEAS-2B epithelial cells treated with PBS, exosomes derived from macrophages treated with PBS. **e** Mitochondrial OCR was determined using a Seahorse XF Extracellular Flux Analyzer in epithelial cells co-cultured with exosomes from macrophages exposed to HIV Tat protein, exosomes from control cells, and exosomal miR-27a mimic for 24 h,12 replicates per group. **f**–**i** Bar graph showing individual mitochondrial function parameters calculated from data in **e**, including Basal respiration, maximal respiration, spare capacity, and ATP production. The data are presented as mean ± SEM from three independent experiments. The Wilcoxon’s test was used to compare between two groups. *p < 0.05 vs. control. **j** Intracellular ATP content, *p *<* 0.05

We next wanted to determine the functional effects of exosomal miR-27a. By using the species-conserved database and TargetScan (Whitehead Institute for Biomedical Research, Cambridge, MA), putative binding sites for miR-27a were identified in the 3′-UTR of peroxisome proliferator-activated receptor gamma (PPARγ), suggesting that increases in miR-27a can reduce PPARγ expression. PPARγ is an important transcriptional regulator of mitochondrial gene expression and plays a critical role in metabolic and inflammatory response. Recently, we have shown that PPARγ plays a pivotal role in epithelial immune function^40^. We first determined the expression of PPARγ in BEAS-2B cells that are exposed to exosomes from macrophages treated with Tat. As shown in Fig. 5a, b, expression levels of PPARγ mRNA and protein were significantly reduced in BEAS-2B cells exposed to exosomes from macrophages exposed to Tat or miR-27a mimic, compared with control cells.

To further confirm the role of miR-27a, we performed additional experiments using PPARγ luciferase reporter constructs. Based on the computationally predicted binding sites of miR-27a on the 3′-UTR of the human PPARγ gene, we developed a luciferase reporter construct as shown in the schematic map (Fig. 5c). Then, through a luciferase activity assay, we validated whether exosomal miR-27a could modulate PPARγ mRNA expression through directly targeting 3′-UTR in epithelial cell line BEAS-2B cells. As shown in Fig. 5d, miR-27a mimic significantly reduced luciferase activity, compared with the scramble miRNA. We next determined whether exosomes secreted from macrophages altered the PPARγ luciferase activity. BEAS-2B cells transfected with PPARγ luciferase constructs for 48 h were exposed to exosomes from Tat- or PBS-treated macrophages. Cells treated with exosomes from Tat-treated macrophages showed a significant reduction in PPARγ luciferase activity compared with cells that were treated with exosomes from PBS-treated macrophages (Fig. 5d).

We next sought to define the functional impact of reduced PPARγ expression on cellular metabolic-mitochondrial response. In order to assess the metabolic response, we measured oxidative phosphorylation (OXPHOS) and glycolysis that contribute to energy production by employing Seahorse assays. HIV Tat directly suppressed oxygen consumption rate (OCR) of human macrophages, as shown in Fig. S3A. Furthermore, we found HIV Tat effectively induced mitochondrial reactive oxygen species (ROS), compared with control cells (Fig. S3b). To determine the effect of exosomal miRNA on mitochondrial function of lung epithelial cells, BEAS-2B cells were co-cultured with exosomes secreted from human macrophages exposed to Tat or PBS. Exosomes secreted by Tat-human macrophages or transfected with miR-27a mimic decreased basal respiration of epithelial cells, in comparison with the control group (Fig. 5e). We also determined the parameters reflecting mitochondrial bioenergetics status, including basal respiration, spare respiratory capacity (SRC), maximal respiration, and ATP turn over, which provided information regarding the mitochondrial respiratory status of recipient cells. As shown in Fig. 5f–i, in comparison with the control groups, exosomes secreted by HIV viral protein-treated macrophages significantly reduced OCR in epithelial cells, blunting oxidative capacity through OXPHOS. Exosomes transfected with miR-27a also showed a similar trend of OCR reduction, similar to exosomes from Tat-treated macrophages. Intracellular ATP assay showed that exosomes from Tat protein-treated human macrophages significantly reduced ATP concentration in epithelial cells, compared with control cells (Fig. 5j).

### Exosomes secreted by macrophages exposed to HIV Tat protein increase glycolysis

**Fig. 6:**
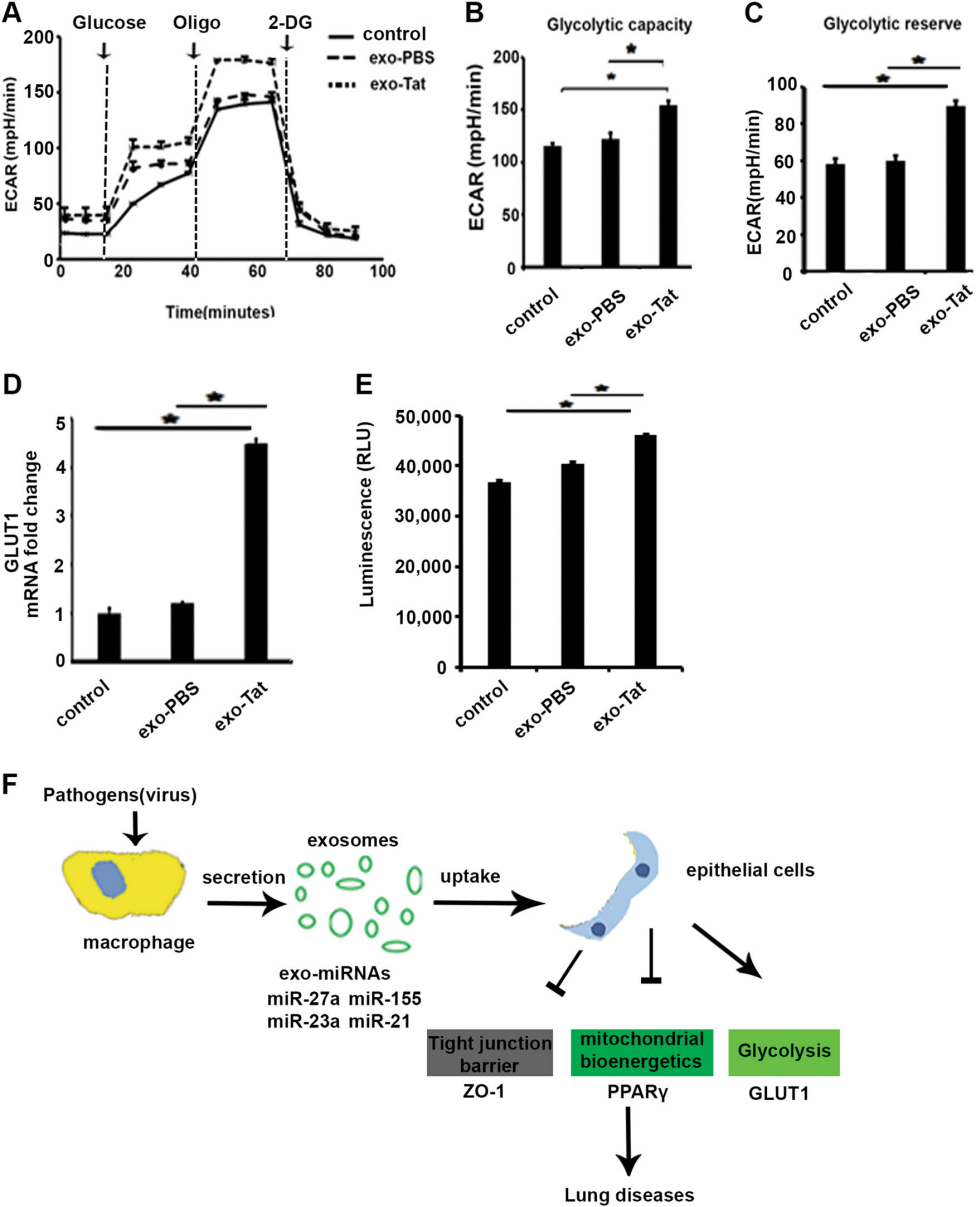
Exosomes secreted by macrophages exposed to HIV protein increase glycolysis in lung epithelial cells. **a** BEAS-2B cells were treated with PBS, exosomes from Tat- or PBS-treated human macrophages, or exo-miR-27a, respectively. Twenty hours later, glycolysis was measured by extracellular flux analysis. Extracellular acidification rate (ECAR), reflective of glycolysis in basal state, glycolytic activity, and corresponding glycolytic reserve (as assessed by the difference between oligomycin A-induced ECAR and basal ECAR), as shown in **b** and **c**. The data are presented as mean ± SEM from three independent experiments. The Wilcoxon’s test was used to compare between two groups. *p < 0.05 vs. control. **d** GLUT1 mRNA expression was detected by RT-qPCR; 18S served as internal control. Student’s test, *p *<* 0.05. **e** Glucose uptake was detected. *p *<* 0.05. **f** Schematic showing cell–cell communications between macrophages and alveolar epithelial cells in alveolar microenvironment during HIV infection. The schematic depicts that exosomal microRNA shuttling from macrophages to epithelial cells can lead to alterations in epithelial barrier proteins and mitochondrial bioenergetics resulting in altered function

We then sought to investigate whether glycolysis is altered in epithelial cells that are exposed to exosomes from Tat-treated macrophage. Extracellular flux analyses were performed on BEAS-2B cells exposed to exosomes from Tat- or PBS-treated macrophages. Tat-treated macrophage-derived exosomes induced an increase of glycolytic activity and glycolytic reserve (Fig. 6a–c), indicating that exosome shuttling stimulates glycolysis in the recipient cells. Finally, we analyzed expression of glycolysis-associated genes. Compared with mock controls, mRNA expression of GLUT1 was significantly increased by exosomes derived from macrophages treated with Tat (Fig. 6d). Glucose uptake assay also demonstrated increased glucose consumption in epithelial cells (Fig. 6e).

Collectively, these data indicate that exosomes secreted from HIV viral protein-treated macrophages can alter cellular metabolism in recipient cells through exosomal miRNAs shuttling, leading to a decrease of OXPHOS and an increase of glycolysis.

## Discussion

It is increasingly recognized that HIV-infected macrophage secrete extracellular vehicles that contribute to HIV immune response and might have a critical role in mediating intercellular communication between immune and structural cells. However, molecular mechanisms underlying cell–cell crosstalk in the setting of HIV infection remain largely undefined. Here we show that macrophages exposed to recombinant proteins Tat or gp120 express high levels of miRNAs, which are involved in inflammatory responses. We found a similar expression of miRNA pattern in macrophage-secreted exosome-isolated BAL fluid of HIV transgenic rats. We investigated the functional impact of miR-23a and miR-27a secreted in exosomes of Tat-treated macrophages. We found that exosomal miR-23a attenuated integrity of tight junction barrier via posttranscriptional modulation of ZO-1 3′-UTR in epithelial cells, whereas exosomal miR-27a altered mitochondrial bioenergetics of recipient cells by targeting mitochondrial respiratory chain protein PPARγ while stimulating glycolysis. These data suggest that exosomal miRNAs shuttling from macrophage to epithelial cells can significantly alter barrier integrity and bioenergetics of the recipient cells, and may play a critical role in immune modulation.

Exosomes may act as regulators of both innate and acquired immunity by stimulating cytokine production, inflammatory responses, and antigen presentation^41^. Several studies have reported effects of HIV infection on exosome secretion and have shown immunomodulatory effects in monocytic cells and macrophages^27,42^. Exosomes released by different cell types contain heterogeneous molecular compositions, which lead to diverse modulation of physiological and pathological processes, including cell growth, cell cycle, metabolism alteration, and immune regulation^43–46^. Notably, exosomes released by HIV-infected cells might modulate the entire tissue microenvironment through delivering molecular content to recipient cells, to help HIV replication, including CD4^+^ T lymphocytes and resident tissue macrophages^27,34^. Exosomes also might play a crucial role in immune cell-based immunotherapy against HIV infection^47^.

However, few studies have examined the link between HIV infection and active exosome secretion in lung cells, as well as the interaction between immune cells and lung epithelial cells. Our data for the first time report that exosomal miRNAs secreted by macrophages modulate lung epithelial response via miR-23a and miR-27a, whose expression was significantly increased in exosomes released by HIV viral protein-treated macrophages. These miRNAs are known to regulate tight junction barrier and mitochondrial function in lung cells. Our studies demonstrate that exosomal miRNAs released from macrophages can lead to a series of events in recipient alveolar epithelial cells, resulting in impairment of tight junction barrier integrity and mitochondrial bioenergetics. These changes in alveolar microenvironment can increase susceptibility to lung infection and injury (Fig. 6f).

MiRNAs have been widely shown to contribute to immune regulation during HIV infection^48,49^. Several studies have focused on cellular miRNA targets in the retroviral genome and their influence on viral replication, latency, gene expression, or infectivity. For example, miR-28, miR-125b, and miR-382 contribute to HIV-1 latency in lymphocytes^50^. Expression of miR-155 has been shown to contribute to HIV inflammatory response and establish latency in T lymphocytes^51^. Our data show that miR-155 and miR-21 are expressed in Tat- and gp120-treated macrophages, and are also secreted in exosomes in vitro and in vivo from HIV transgenic rats, and may contribute to immune dysregulation in HIV-infected individuals.

Previously we identified and published that primary alveolar epithelial cells from HIV-Tg had significant barrier dysfunction^52^. Intercellular tight junctions of the epithelium are crucial for the formation of epithelial barrier and decrease in TER is correlated with disruption of tight junction proteins (claudin 1, 2, 4, occludin, and ZO-1) with increased permeability^53^. Growing evidences indicate that lung epithelial damage results in impairment of the tight junction barrier, which disrupts homeostasis of the tissue microenvironment. Junctional adaptor protein ZO-1 has been reported to play a central regulatory role in epithelial barrier formation^54,55^. In this study, we show that crosstalk between HIV-infected macrophages and lung epithelial cells impairs epithelial barrier integrity through transcellular delivery of exosomes. Our data show that macrophage-derived exosomal miR-23a can alter epithelial barrier by targeting ZO-1 in epithelial cells. Conversely, miR-23a inhibition in macrophages exposed to HIV viral protein restores the epithelial barrier integrity of epithelial cells through upregulation of ZO-1 expression. These data indicate that HIV-infected macrophages modulate epithelial barrier integrity by secreting exosomal miRNAs.

Mitochondrial dysfunction in host target cells is increasingly recognized to contribute to pathogenesis of immune dysfunction in HIV infection. Moreover, antiretroviral agents have been associated with serious adverse reactions, some of which result from mitochondrial dysfunction^56^. However, the mechanisms of mitochondrial dysfunction in HIV infection have not been elucidated. As our data showed an increased expression of miR-27a in macrophages, which regulates key mitochondrial proteins, we hypothesized that exosomal miR-27a might result in mitochondrial dysfunction of recipient epithelial cells. As a target of miR-27a, PPARγ is an important transcriptional regulator of mitochondrial gene expression and plays a critical role in metabolic and inflammatory response. Further, we and others have shown that PPARγ plays an important role in host defense against infections^57,58^. In the present study, we show that exosomes released from macrophages treated with HIV viral protein significantly reduce expression of PPARγ in recipient epithelial cells. Most importantly, the functional impact of reduced PPARγ expression include alterations in mitochondrial bioenergetics, such as reduced oxygen consumption rate and SRC with increased glycolysis in recipient epithelial cells. These data for the first time show that exosomal miRNAs released from macrophages exposed to HIV viral proteins can cause mitochondrial dysfunction in the recipient cells.

Emerging data indicate that exosomes are involved in cell–cell crosstalk during infectious and inflammatory diseases. In the tissue microenvironment, there are a broad range of miRNAs, which contribute to both physiological and pathological responses. Through bioinformatics assays, our studies focused on selective miRNAs. Our data show that excessive expression of exosomal miR-23a, miR-27a, miR-155, and miR-21 released by HIV viral protein-treated macrophages disrupt tight junction barrier integrity and mitochondrial biogenesis of epithelial cells. Recent studies show that exosomal miRNA expression levels are significantly increased in patients’ sera and might modulate interaction between HIV-infected immune cells and tissue microenvironment. Our data are the first to show that exosomes released from macrophages exposed to HIV viral proteins modulate epithelial barrier integrity and bioenergetics of the recipient cells. These findings may have potential implications in correction of immune dysfunction in HIV-infected individuals through targeting miRNAs or target genes, such as PPARγ. Blockade of intercellular communication within lung microenvironment may lead to novel interventional strategies for immune modulation in HIV. Further research is needed for better understanding exosomal miRNAs in the pathogenesis and immune regulation of HIV.

## Materials and methods

### Chemicals and reagents

Recombinant HIV IIIB Oligomeric Glycoprotein gp120 (Baculovirus) (product#1061) and Recombinant Tat HIV-1IIIB (product#1002) were obtained from ImmunoDX (USA). The 100 ng/ml dose of HIV-1 Tat and gp120 proteins was selected from dose–response experiment. Antibodies against CD9, CD63, ZO-1, α-tubulin, and actin were obtained from Cell Signaling Technology (USA); anti-PPARγ (E8)(sc-7273), TSG101, and all other antibodies were purchased from Santa Cruz Biotechnology (USA). miRNA-23a mimic and antagomir, miRNA-27a mimic and antagomir were purchased from Thermo Fisher Scientific (USA).

### Ethics statement

The source of human monocytes was whole blood of healthy donors and the study (Healthy Donor protocol, IRB# 58507) was approved by the Ethics committee and the Institutional Review Board of Emory University, and informed written consent was obtained from all donors. All human subjects were adults.

All animal procedures were reviewed and approved by the Institutional Animal Care and Use Committee (IACUC) at Atlanta VA Medical Center and the approval IACUC number is V021-17. All animal experiments in this study conform to the IACUC Guidelines as well as National Institutes of Health (NIH) and USDA policies on care and use of animal in research and teaching.

### Human MDM preparation and infection

Isolation of monocytes and culture of macrophages were performed as previously described^59,60^. Briefly, mononuclear cells were isolated by Ficoll-Hypaque Plus (GE Healthcare) gradient, cultured in Dulbecco’s modified Eagle medium (DMEM) supplemented with 10% heat-inactivated fetal bovine serum (FBS) (HyClone, USA) and 50 ng/ml human M-CSF (R&D Systems, USA). Seven days later, purity of macrophages was assessed by flow cytometry ( > 90% CD14^+^) as described previously^33,60^.

MDMs were infected with or without HIV-1 viral particle for 8 days, as described previuosly^33^. This protocol was approved by Emory University Institutional Review Board.

### Isolation of rat alveolar macrophages

HIV-1 Tg and WT Fischer 344 rats were purchased from Harlan Laboratories (Indianapolis, USA)^37^. Alveolar macrophages were isolated from rats as previously described^61^. Rats were asphyxiated with CO_2_; tracheas were cannulated and lungs were lavaged in situ with sterile pyrogen-free physiological saline, and gently withdrawn with a 1 ml tuberculin syringe. Lung lavage fluid was centrifuged at 1000 *×* *g* for 10 min, then supernatant was used to isolate BAL fluid-derived exosomes, and cell pellets were suspended with DMEM/F-12 medium (Thermo Fisher Scientific, USA) for following studies.

### Isolation, characterization, labeling, and purification of exosomes

Exosomes were extracted from rat BAL fluid and cell culture media through ultracentrifugation^62^. An aliquot of the exosomes was visualized using TEM (Experimental Pathology Laboratory Core, Emory University). A separate exosome sample was used to examine exosome markers by western blotting with the following primary antibodies against CD63 and CD9 (Santa Cruz Biotechnology, USA). Through DLS assay, the size of exosomes was determined by NanoPlus-zeta/nanoparticle analyzer (Micromeritics, USA) and NTA was performed to detect size and concentration of particles through NanoSight NS300 (Malvern, UK). Exosomes were labeled with PKH67 through PKH67 Green Fluorescent Cell linker Mini Kit for General Cell Membrane Labeling (Millipore Sigma, USA), then labeled with Acridine Orange (Thermo Fisher, USA), and finally purified with Exosome Spin Columns (MW3000) (Invitrogen, USA), as previously described^62^.

### Cell culture and treatment

THP-1 cells were obtained from American Type Culture Collection (ATCC, USA) and maintained in RPMI1640 (Corning Cellgro, USA) containing 10% FBS (Hyclone, USA), and BEAS-2B cells (ATCC, USA) were grown in endothelial cell basal medium (LONZA, USA) containing 10% FBS. THP-1 cells were treated with 10 µmol/l PMA (Millipore Sigma, USA) for 1–3 days. THP-1-derived macrophages and human MDM were transfected with antagomir against miR-23a, miR-23a mimic, antagomir against miR-27a, miR-27a mimic, Cy3™ Dye-Labeled Pre-miR Negative Control #1, or Cy3-PreCy3 dye-labeled anti-miR-negative inhibitor control (Thermo Fisher, USA) through Lipofectamine 3000 reagent (Thermo Fisher, USA) according to the manufacturer’s instructions. Cells were incubated with RNA complexes for 48–72 h before analysis. For in vitro experiments, cells were treated with recombinant HIV viral proteins Tat (100 ng/ml) and gp120 (100 ng/ml). Control cells were treated with sterile PBS. Exo-Fect Exosome transfection kit (SBI, USA) was used to convert miRNA mimic and scramble into exosomes for transfecting recipient cells.

### Preparation of RNA and miRNA-qRT-PCR

RNA preparation and qRT-PCR were performed, as previously described^32^. PCR primers and PCR mix were purchased from Thermo Fisher Scientific (USA). Each sample was analyzed in triplicate, using an ABI PRISM 7500 quantitative PCR apparatus (Thermo Fisher Scientific) and the expression values were normalized against the small housekeeping RNAs U6 snRNA, 18S, or GAPDH.

### Western blotting analysis

Western blotting analysis was performed as described previously^63^. Collected cells and exosomes were lysed with RIPA buffer (Millipore Sigma, USA) containing 1% Triton X-100 and protease inhibitors.

### Immunofluorescence

Immunofluorescence was performed as described previously^33^. Primary antibody anti-ZO-1 was obtained from Thermo Fisher (USA). First, slides were incubated overnight at 4 °C, then incubated with the appropriate Alexa Fluor 488-goat anti-rabbit (Invitrogen, A11008) secondary antibody, and mounted with Vectashield containing DAPI (Vector Laboratories, H-1200, USA). All slides were visualized by using Olympus FV1000 confocal microscope (Japan).

### Dual-luciferase assay

pMirTarget, pMirTarget-wt-PPARγ-3′-UTR, and pMirTarget-wt-ZO-1-3′-UTR were obtained from OriGene Technologies (USA). By using Lipofectamine™ 3000 (Invitrogen, USA), BEAS-2B cells were transfected with the miR-27a mimic and miR-23a mimic, as well as Cy3-dye-labeled negative pre-miR control (Thermo Fisher Scientific, USA), and reporter vectors. A Dual-Luciferase Assay kit (Promega, USA) was used to detect luciferase activity according to the manufacturer’s instructions.

### Extracellular flux analysis

The bioenergetic flux was assessed using the Seahorse XF96 extracellular flux analyzer (Seahorse Biosciences, USA), as described previously^32^. The cells were plated at 2 × 10^4^ cells per well and treated with exosomes. Agilent XF cell Mito stress kit (Agilent Seahorse Biosciences, USA) was used to measure the OCR and extracellular acidification rate (ECAR). Agilent Seahorse XF Glycolysis Stress Test was used for measuring glycolytic effects. OCR and ECAR were then determined using the XF96 plate reader as recommended by the manufacturer and calculated by the Seahorse Wave software (Agilent Seahorse Bioscience, USA).

### Glucose uptake

The cells were seeded at a density of 2 × 10^4^ cells per well in 96-well plates, then incubated with 1 mM 2-deoxyglucose for 2 h. After washing with PBS, the levels of glucose uptaken by cells was measured with a Glucose Uptake-Glo^TM^ Assay Kit (Promega, USA) according to the manufacturer’s protocol. Relative fluorescence units were determined via a Synergy™ 2 Multi-Detection Microplate Reader (BioTek, USA).

### ATP production

To determine the intracellular ATP production, 1 × 10^6^ cells were collected and intracellular ATP concentration was measured through ATP Determination Kit (Thermo Fisher, USA) according to the manufacturer’s protocol.

### Statistical analysis

Statistical analyses were performed using GraphPad Prism software version 5.0 (GraphPad Software). All experiments were repeated at least three to five times. Data are expressed as mean ± SEM, unless specified. Student’s *t*-tests were used for two-group comparisons, one-way analysis of variance with Bonferroni post tests was used for multiple group comparisons, and *p*-value < 0.05 was considered significant.

## Supplementary information

Supplemental data
