# Supplementary material for: Macrophages exposed to HIV viral protein disrupt lung epithelial cell integrity and mitochondrial bioenergetics via exosomal microRNA shuttling
Source: Cell Death Dis. 2019 Aug 2;10(8):580. doi: 10.1038/s41419-019-1803-y (PMC6675785; doi:10.1038/s41419-019-1803-y)

**Supporting Figure Legends**

**FigureS1** Co-culture with macrophages exposed to HIV viral Tat protein impairs integrity of tight junction barrier in lung epithelial cells. Human macrophages were treated with Tat(100ng/ml) for 24 hours prior to co-culturing with lung epithelial cells Beas-2B cells, as shown in Fig.S1a. After 24 hours of co-culture, expression of tight junction protein ZO-1 were detected via q-PCR and western blotting, as shown in Fig.S1b-1c. **P＜ 0.05.*

**Figure S2** Recipient BEAS-2B cells were transfected with pMirTarget control vector and Cy3-dyed negative pre-miR control( Thermo Fisher, USA), non-labeled negative pre-miR was used as negative control. 24 hour later, transfection efficiency was clarified via fluorescence microscopy. **P＜ 0.05.*

**Figure S3** HIV viral Tat protein suppresses OCR and increases mitochondrial ROS in human macrophages. (a) Human macrophages were treated with Tat (100ng/ml) for 24 hours, and mitostress assay was performed. (b) MitoSOX Red (Thermo Fisher, USA) was added to final concentration of 5µM according to manufacturer’s protocol. Representative confocal images of human macrophages showing in increase in mitochondrial MitoSOX fluorescence following treatment with Tat(100ng/ml) for 24 hours.

Supplementary Figure1.


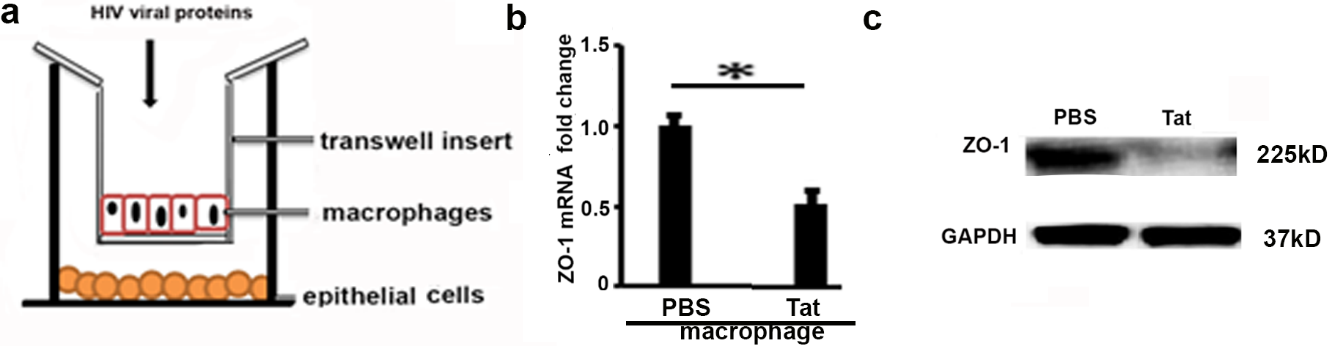


Supplementary Figure 2.


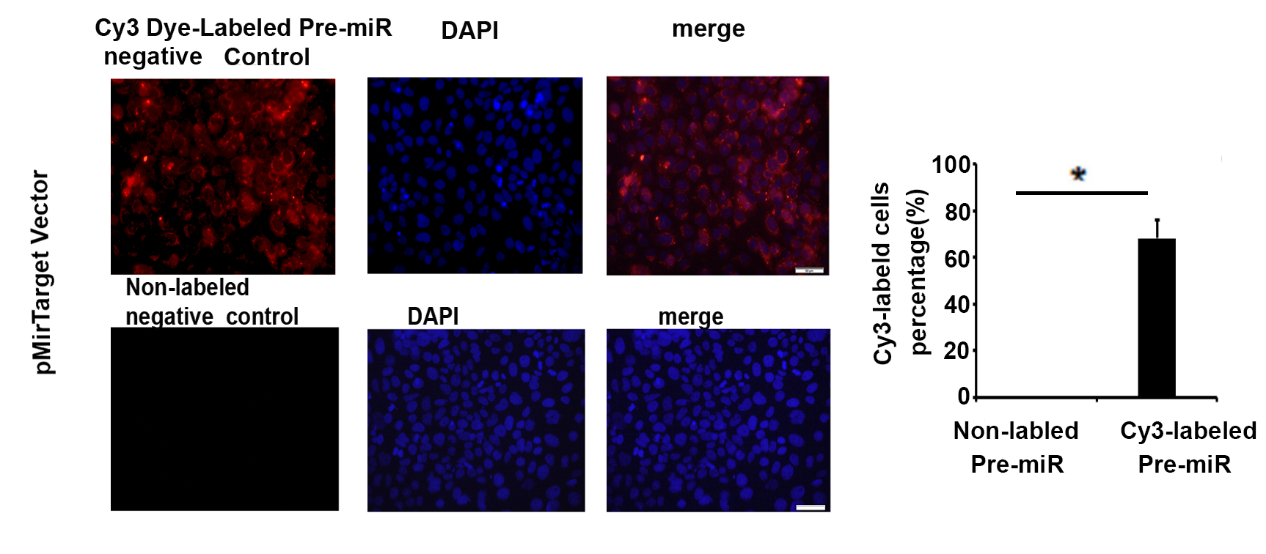


Supplementary Figure 3.


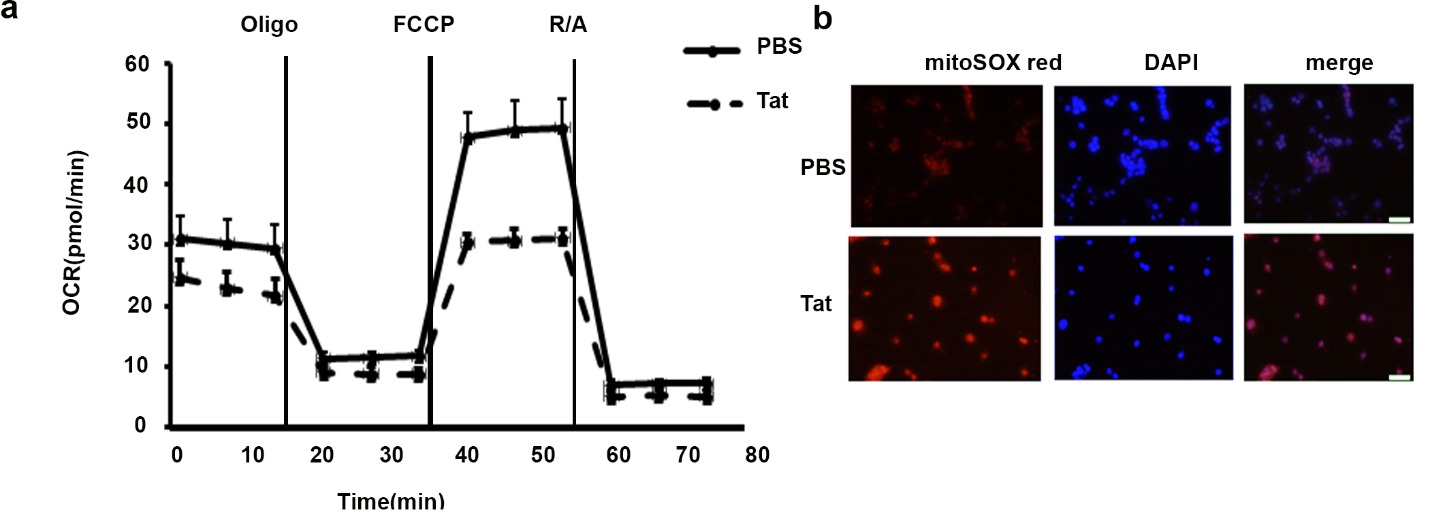

Supplement: Supplementary file 1 — Supplemental data [file 41419_2019_1803_MOESM1_ESM.docx]
